# Environmentally Friendly Polymers and Polymer Composites

**DOI:** 10.3390/ma13214892

**Published:** 2020-10-31

**Authors:** Rafael Balart, Nestor Montanes, Franco Dominici, Teodomiro Boronat, Sergio Torres-Giner

**Affiliations:** 1 Technological Institute of Materials (ITM), Universitat Politècnica de València (UPV), Plaza Ferrándiz y Carbonell 1, 03801 Alcoy, Spain; nesmonmu@upvnet.upv.es (N.M.); tboronat@dimm.upv.es (T.B.); 2 Civil and Environmental Engineering Department, University of Perugia, UdR INSTM, Strada di Pentima 4, 05100 Terni, Italy; franco.dominici@unipg.it; 3 Research Institute of Food Engineering for Development (IIAD), Universitat Politècnica de València (UPV), Camino de Vera s/n, 46022 Valencia, Spain

**Keywords:** bio-based polymers, biodegradable polyesters, green composites, wood plastic composites, natural additives and fillers, composites characterization, bioplastics manufacturing

## Abstract

In the last decade, continuous research advances have been observed in the field of environmentally friendly polymers and polymer composites due to the dependence of polymers on fossil fuels and the sustainability issues related to plastic wastes. This research activity has become much more intense in the food packaging industry due to the high volume of waste it generates. Biopolymers are nowadays considered as among the most promising materials to solve these environmental problems. However, they still show inferior performance regarding both processability and end-use application. Blending currently represents a very cost-effective strategy to increase the ductility and impact resistance of biopolymers. Furthermore, different lignocellulosic materials are being explored to be used as reinforcing fillers in polymer matrices for improving the overall properties, lower the environmental impact, and also reduce cost. Moreover, the use of vegetable oils, waste derived liquids, and essential oils opens up novel opportunities as natural plasticizers, reactive compatibilizers or even active additives for the development of new polymer formulations with enhanced performance and improved sustainability profile.

The demand for plastics has remarkably increased in recent decades and pollution deriving from these materials has become one of the most prominent environmental concerns of recent years. Moreover, conventional polymers are obtained from fossil fuels and show high persistence in the environment, which results in sustainability issues related to petroleum depletion and waste management. Recent regulations on the recyclability of materials and environmental requirements have compelled manufacturers and research institutions to develop environmentally friendly polymers and polymer composites in the last years. This Special Issue compiles one review and fifteen articles written by researchers and technologists that reflects the growing interest in the development of sustainable materials that offer the possibility of rendering interesting applications in food packaging as well as in other sectors such as pharmaceutics, agriculture or biomedicine.

In this context, the biopolymers obtained from renewable resources currently represent a sustainable alternative to petroleum derived polymers and they can also contribute to decrease the product carbon footprint. The next generation of biopolymers will be synthesized from non-edible and highly available plants and also from agro-food and industrial wastes or by-products, which contribute to the progress of the Circular Bioeconomy. Furthermore, some biopolymers are also biodegradable and their resultant articles can be disintegrable in controlled compost soil. These materials include different types of carbohydrates, for example thermoplastic starch (TPS), cellulose and its derivatives, alginates, pectin, chitin or chitosan, animal-based proteins, such as silk, gelatin or collagen, and plant-based proteins as well as lipids. In this regard, the review performed by Mellinas et al. [1] gathered the most recent studies regarding the sources, different types, structure, and potential uses of pectin. This natural-based polysaccharide is currently extracted from plants and thereafter isolated as a bioplastic material. However, by using innovative methods, pectin can also be obtained from different kinds of waste biomass, ranging from the by-products of juice manufacturing to the peels and seeds of orange, mango, banana, lime or pomegranate. Authors also showed that the final applications of pectin can be very diverse due to the variability in its structure, being very promising in food packaging in the development of active systems.

Chitin and chitosan are also relevant examples of polysaccharides showing a great deal of potential in pharmaceutical and biomedical applications. Chitin is a *β*-(1,4)-*N*-acetyl-d-glucosamine found in the shells of crabs, lobsters, and shrimps. Chitosan, which is obtained from the alkaline *N*-deacetylation of chitin, is a cationic polysaccharide composed of a linear chain of d-glucosamine and *N*-acetyl-d-glucosamine linked via a *β*-(1,4) bond. Both carbohydrates are largely present in marine fishery by-products and are also known to exhibit several active and bioactive properties. For instance, in the research article of Taokaew et al. [2], chitin was extracted from local snow crab shell waste and used to produce microgels with sizes ranging from 5 to 200 μm using a batch process of emulsification and gelation. Chitin microgels with narrow size distribution with an average size as low as 7 μm and porous spherical morphology were successfully achieved at chitin contents of 3 wt %, exhibiting pH-dependent swelling-shrinking behavior for pH values between 2 and 10. In another study, Ahlawat et al. [3] reported the antioxidant effect on a human SH-SY5Y neuroblastoma cell line of chitosan nanoparticles synthesized using an ionotropic gelation method with tripolyphosphate (TPP) as the cross-linking agent. Chitosan particles with an average size of ~200 nm showed reduced rotenone-initiated cytotoxicity and apoptotic cell death. According to the authors, these novel chitosan nanoparticles might be a neuroprotective agent for the prevention of Parkinson’s disease.

The main drawbacks of most biopolymers are their inferior thermal resistance and poorer mechanical properties when compared to conventional polymers such as polyolefins and styrene-based polymers. In the case of naturally occurring polymers, they habitually show strong hydrophilic character, being highly affected by water. All these characteristics limit the expansion of biopolymers to food packaging and other commodity areas. Blending represents a very cost-effective solution to overcome or, at least, minimize the low ductility and toughness of biopolymers. For instance, bacterial cellulose (BC), which is mainly produced by the bacteria of genera Acetobacter, such as *Acetobacter xylinum*, can result in strong and high-barrier films composed of interconnected networks of cellulose nanofibers. Since it shows low-breaking elongation, Potivara and Phisalaphong [4] achieved a ~4-fold improvement in the tensile strength and elongation at break of BC films by the incorporation of natural rubber (NR). When BC pellicles were immersed in a diluted NR latex (NRL) suspension at 2.5–5.0% dry rubber content (DRC) in the presence of ethanol aqueous solution at 50–60 °C, the resultant NR/BC films showed improved resistance to water and also high resistance to non-polar solvents such as toluene, biodisintegrating completely in soil after 5–6 weeks. Among biopolyesters, polylactide (PLA) is already well positioned in the bioplastics market due to its good processability, balanced properties, relatively low price, and industrial compostability. Despite this, PLA materials are very brittle and for this reason Lascano et al. [5] developed binary blends of PLA with the petrochemical biodegradable copolyester poly(butylene succinate-*co*-adipate) (PBSA) and an epoxy styrene-acrylic oligomer (ESAO) as a reactive compatibilizer. The compatibilized blend containing 20 wt % of PBSA showed elongation-at-break values of approximately 121%, whereas the addition of 30 wt % of PBSA also improved the impact strength in V-notched samples from 2.48 to 5.75 kJ/m^2^. Furthermore, the addition of 10 wt % PBSA slightly improved the shape memory recovery of PLA. With the same scope, ternary blends of PLA with 40 wt % of different poly(ε-caprolactone) (PCL) and TPS combinations were reported by Quiles-Carrillo et al. [6]. Although all the biopolymer blends were immiscible, the combination of PLA with 30 wt % of PCL and 10 wt % of TPS showed an elongation at break as high as 196.7%, which was approximately 40 times higher than that of neat PLA. The resultant improvement in toughness was ascribed to the “island-and-sea” morphology of the PLA-based blends, where the rubber-like PCL phase was finely dispersed in the form of micro-sized spherical domains favored by the co-presence of TPS.

Despite the high suitability of biopolymers as candidates for sustainable applications, they are still not cost-effective. This is particularly true for polyhydroxyalkanoates (PHAs), a family of linear polyesters produced in the nature by the action of bacteria during the fermentation of sugar or lipids in famine conditions. The use of biomass derived from food processing by-products and agro-food wastes does not only represent a possible strategy to reduce price but also allows to achieve a more sustainable material concept since they valorize large amounts of residues. Moreover, natural fillers offer several advantages such as improved biodegradability, low cost, low abrasion, high specific strength, low density, etc. Furthermore, when a bio-based and biodegradable polymer is combined with natural fillers, the resultant material was named “green composite”, meaning that the whole material was obtained from renewable resources and is biodegradable. In this context, Melendez-Rodriguez et al. [7] developed green composites based on different blends of commercial poly(3-hydroxybutyrate) (PHB) and purified poly(3-hydroxybutyrate-*co*-3-hydroxyvalerate) (PHBV), which was produced by mixed microbial cultures (MMCs) using biomass derived from fruit pulp waste, filled with 10 wt % rice husk flour (RHF). The green composites were prepared using triglycidyl isocyanurate (TGIC) as a compatibilizer and dicumyl peroxide (DCP) as an initiator. The incorporation of the biosustainably produced PHBV at contents of 5–10 wt % counteracted the stiffness and fragility induced by RHF, yielding films with a balanced performance in terms of strength and ductility. The resultant films also showed improved thermal stability, thus having a larger processing window, and also a medium and high barrier to water vapor and aroma, respectively, being potential candidates for rigid packaging applications.

Biopolymers are also excellent candidates for manufacturing wood plastic composites (WPCs) and natural fiber-reinforced plastics (NFRPs) in combination with plant fibers, for instance flax, kenaf, ramie, jute, hemp or sisal. The mechanical behavior of composite materials can be, however, influenced by several environmental factors, for example temperature, humidity, radiation or chemical agents, as well as the type, variation in time, loading speed, direction or duration of the mechanical stresses to which they are subjected. As reported by Bolcu and Stănescu [8], an additional important aspect concerning the mechanical properties of polymer composite materials is given by the non-uniformities that appear in the technological process of fabrication. In the case of NFRPs, the main factor influencing their mechanical behavior is the uneven distribution of the fibers in matrix. In this research study, it was studied the influence of material irregularity on the mechanical behavior of hemp-reinforced composite materials produced with natural Dammar, a gum resin obtained from trees of the family *Dipterocarpaceae*, mixed with an epoxy resin. Authors concluded that the analyzed material was uniform and, in the case of the composites, the analyzed material showed reinforcing material proportions, resin specifically, different from those of the reference materials. According to this, when considering the non-uniformity degree, the resin transfer, the structural reactions, and the interface effects are phenomena that should be taken into account.

Inorganic fillers, such as nanoclays, or different types of nanoparticles, can also be used to enhance the flame retardancy and thermal stability of biopolymers and their NFRPs. In this context, Khalili et al. [9] developed natural fiber-reinforced PLA laminates by a conventional film stacking method using biopolymer films and natural fabrics with different cross-ply layups followed by hot compression. Natural fiber composites of PLA filled with varying concentrations of nanoparticles of hydroxyapatite (nHA) were produced by the same manufacturing technique. The flame behavior of the PLA/nHA composites was evaluated by the UL-94 test, demonstrating that only the composite containing the highest quantity of nHA, that is, 40 wt %, achieved an FH-1 rating and exhibited no recorded burn rate, whereas other composites obtained only an FH-3. Moreover, upon the addition of nHA, the thermal analysis indicated that the mass residue was improved by 279% whereas the thermal decomposition and the mass loss rate was also enhanced slightly. In another study, Larraza et al. [10] produced graphene oxide (GO), which was thereafter subjected to different levels of exfoliation by sonication and centrifugation and finally used to reinforce sodium alginate (SA). The latter biopolymer is a hydrophilic linear polysaccharide composed of (1→4)-*β*-D-mannuronic acid and (1→4)-*α*-l-guluronic acid units that can be extracted from brown algae (*Macrocystis pyrifera*). Due to restrictions in SA chains motion as a consequence of interactions with GO, the resistance to thermal degradation and mechanical properties of the biopolymer nanocomposites were increased. In particular, the loading of 8 wt % of GO led to an improvement of 65.3% and 83.3% for the tensile strength and Young’s modulus, respectively. According to the authors, the resultant biopolymer nanocomposites show potential applications in pharmaceutics, biomedicine, food industry, etc.

Natural oils and, in particular, vegetable oils, represent a new generation of renewable materials that can positively contribute to progress in the preparation and commercialization of sustainable plasticizers. These are polymer additives that act as internal lubricants, favoring polymer chain mobility and enhancing processability and ductility. Some vegetable oils are also interesting from a chemical point of view due to their triglyceride structure based on a glycerol basic structure that is bonded through esters to fatty acids. They present a different number of unsaturations, which constitute the base for a chemical modification to provide the desired multi-functionality. Thus, selectively modified vegetable oils have been developed as novel renewable materials for the compatibilization of polymer blends and green composites. On this topic, Dominici et al. [11] evaluated the use of maleinized (MLO) and epoxidized (ELO) linseed oils in contents of up to 15 wt % as potential bio-based plasticizers for improving the toughness of Arboform^®^, a lignin/natural fiber commercial composite. It was observed that the addition of ELO at 2.5 wt % improved the impact-absorb energy from 5.4 to 11.1 kJ/m^2^, while a similar improvement of 118% was obtained by the addition of 5 wt % of MLO. Furthermore, both MLO and ELO improved the thermal stability and tensile strength due to the formation of ester bonds between the multiple maleic and epoxy groups present in the oils and the hydroxyl groups of the matrix. In another study, Gonzalez et al. [12] reported the efficiency of MLO as compatibilizer in PLA/diatomaceous earth (DE) at a filler content of 10 wt %. Above five parts per hundred resin (phr) of MLO, the ductile properties were remarkably improved and the impact strength increased to nearly 22 kJ/m^2^, which was almost double the value of the uncompatibilized PLA/DE composite. Similarly, in a first study, Liminana et al. [13] prepared green composite pieces of poly(butylene succinate) (PBS), a petrochemical biodegradable homopolyester, with varying loadings of almond shell flour (ASF) by extrusion with MLO and subsequent injection molding. The MLO content was kept constant at 10:1.5 (wt/wt) in relation to ASF and the optimal formulation in terms of the mechanical properties and filler content was attained for pieces filled with 30 wt % of ASF and containing 4.5 wt % of MLO. In a second work, Liminana et al. [14] used the previous green composites to study the effect of varying the MLO content for a constant ASF loading of 30 wt %. In the composition range from 2.5 to 10 wt %, MLO successfully plasticized the green composites and also acted as a compatibilizer by the reaction of the maleic anhydride pendant groups with the hydroxyl groups of both PBS end chains and cellulose and hemicelluloses of ASF. This compatibilizing effect was observed by a reduction of the gap between the ASF particles and the surrounding PBS matrix and also by the increase in the glass transition temperature (T_g_) of PBS from −28 to −12 °C after the addition of 10 wt % of MLO. According to the authors, the developed green composites can reduce the overall current cost of biopolyesters by using large amounts of waste coming from the almond industry, leading to WPCs with a wood-like color.

Other studies have also been focused on the use of renewable and waste derived liquids for green composite formulations. For example, Araújo et al. [15] employed a by-product of the cashew agricultural industry, termed cashew nut shell liquid (CNSL), as a third component to polypropylene (PP) and high-impact polystyrene (HIPS) blends and studied its effect as a compatibilizer. Technical grade CNSL is indeed a natural lipid that consists of a mixture of phenols in which its major constituent is cardanol. The addition of 2 and 5 phr of CNSL in PP/HIPS blends successfully led to a size reduction of the HIPS domains, which ultimately facilitated the tension transfer from one phase to the other as well as the stabilization of the blended morphology. As a result, the industrial use of CNSL can be regarded as a cost-effective and sustainable compatibilizer in polymer blends since it is derived from waste and is also abundant. Alternatively, essential oils are natural mixtures of compounds that, being completely safe for both humans and the environment, show antimicrobial and antioxidant properties. In the study of Thanh et al. [16], marjoram (*Origanum majorana* L.) essential oil was incorporated into a polyamidoamine dendrimer generation 4.0 (PAMAM G4.0) for the development of a novel and sustainable nanocide against the plant disease *Phytophthora infestans*. The resulting essential oil-containing PAMAM G4.0 showed higher antifungal activity in comparison with PAMAM G4.0 and marjoram volatile oil prepared using the same concentration. The enhanced antimicrobial activity was due to the restricted evaporation of the essential oil in the encapsulating systems, which suggests that the encapsulation of marjoram oil in PAMAM G4.0s can be useful in combating late blight in tomatoes and the use of marjoram oil is very promising in the field of plant disease control as a fungicidal alternative to chemical pesticides in agriculture.

In summary, the Special Issue *Environmentally Friendly Polymers and Polymer Composites* compiles the most recent research works in biopolymers, their blends and composites, and the use of natural additives, such as vegetable oils and other renewable and waste derived liquids, with a marked environmental efficiency devoted to developing novel sustainable materials. The research studies gathered in the present Special Issue can certainly help to reveal the real potential of these materials in different applications. It is also highly expected that they will contribute and trigger the transfer of the current knowledge to industry, particularly in the food packaging sector, which is currently requesting the use of renewable and biodegradable materials to reduce the dependence of polymers on fossil fuels and mitigate the effect of plastic wastes on the environment. However, further studies on biopolymers and the optimization of their processes will be still needed to better control the development of the resulting environmentally friendly materials.
